# Supplementary material for: Phosphoproteomic investigation of targets of protein phosphatases in EGFR signaling
Source: Sci Rep. 2024 Apr 4;14:7908. doi: 10.1038/s41598-024-58619-1 (PMC10995159; doi:10.1038/s41598-024-58619-1)
Supplement: Supplementary file 1 — Supplementary Figures. [file 41598_2024_58619_MOESM1_ESM.pdf]

Supplementary Figures

Phosphoproteomic investigation of targets of protein phosphatases in EGFR signaling

Akihiro Eguchi[1], Jesper V. Olsen[1]

[1]Novo Nordisk Foundation Center for Protein Research, University of Copenhagen, Denmark

For correspondence: akihiro.eguchi@cpr.ku.dk, jesper.olsen@cpr.ku.dk

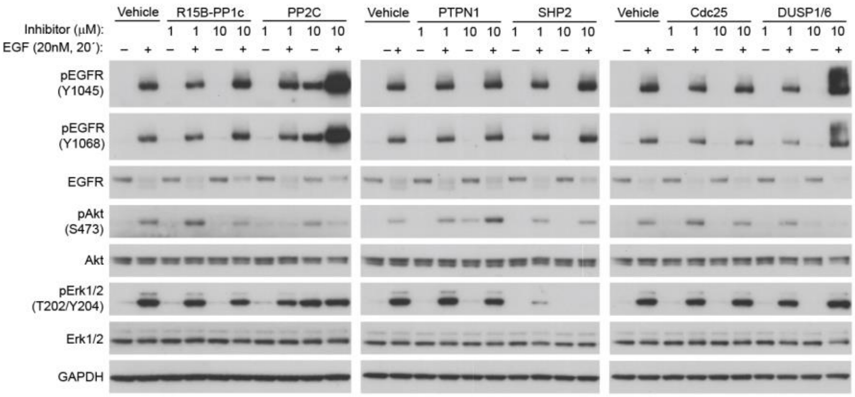

Figure S1. Western blotting analysis using the cell lysates of Hela cells pre-treated with the inhibitors (0.1 or 1 μM) for 15 min followed by 20 min incubation with EGF (20 nM).

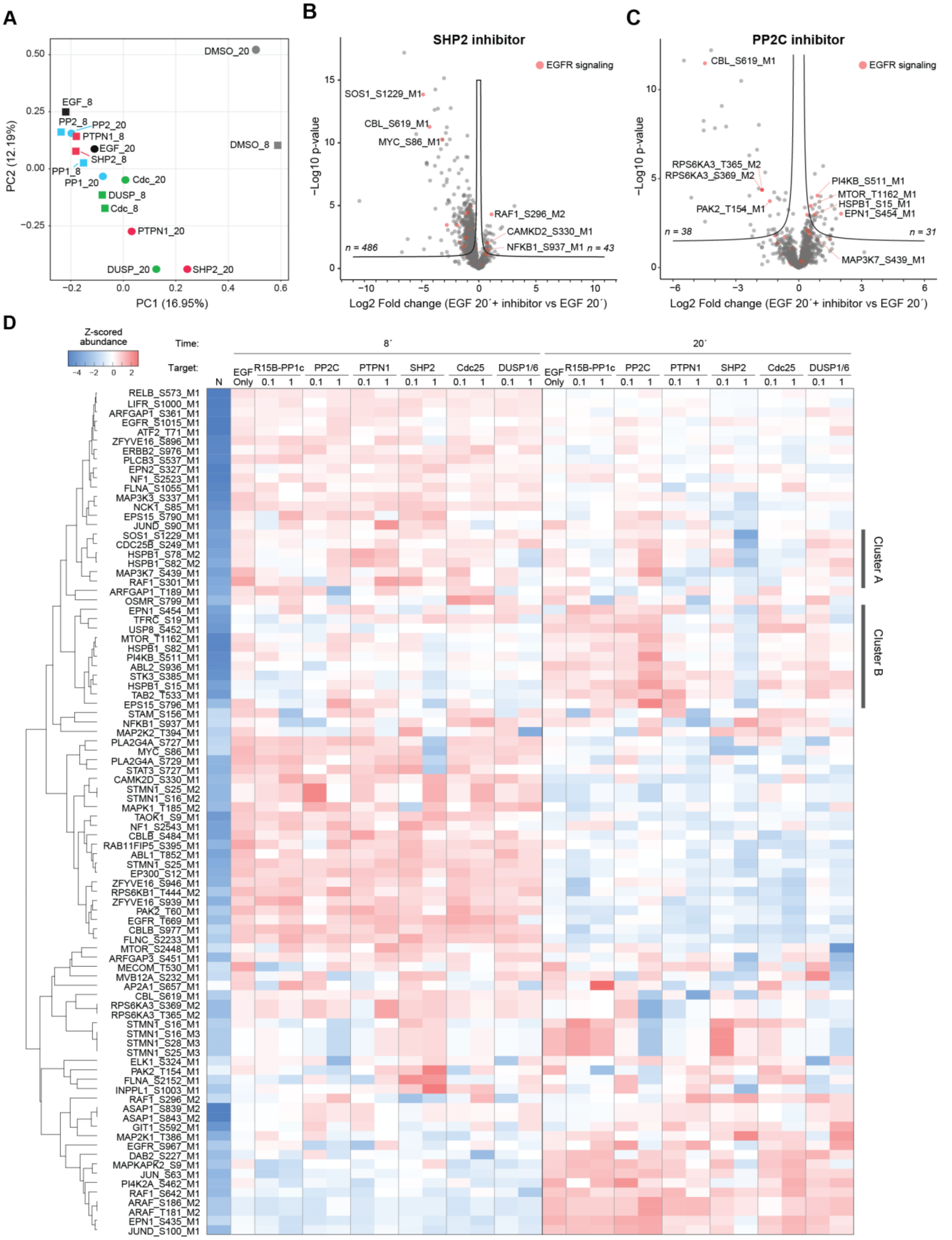

Figure S2. (A) PCA of the log2-transformed abundance of phosphosites. (B, C) Volcano plots highlighting EGFR signaling-related proteins (red). Fold change represents EGF (20 nM, 20´) with the inhibitor of either (B) SHP2 or (C) PP2C. (D) Heatmap showing the z-scored phosphosite abundance.

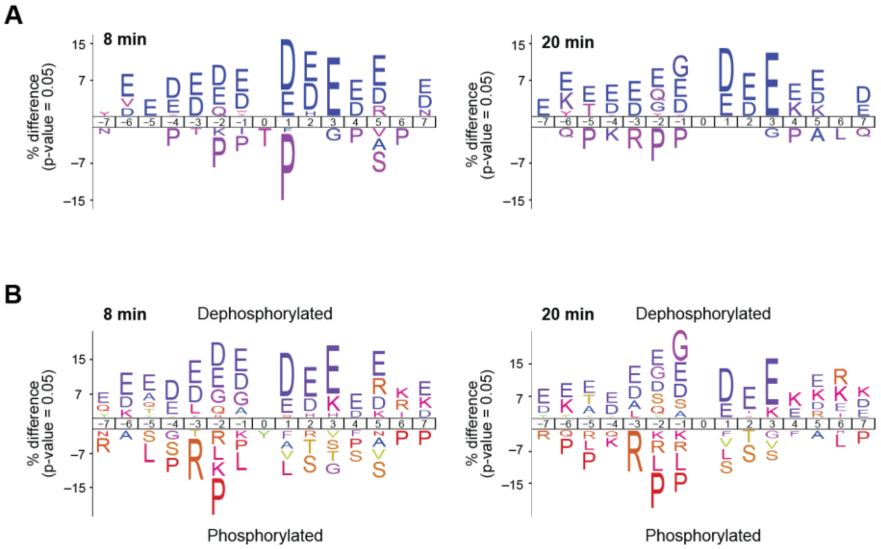

Figure S3. (A, B) Sequence motif enrichment analysis of 15 residues surrounding the regulated phosphosites. Analyses were performed comparing (A) EGF-dependent *de*phosphorylated sites and all the other quantified sites or (B) EGF-dependent *de*phosphorylated sites and phosphorylated sites.

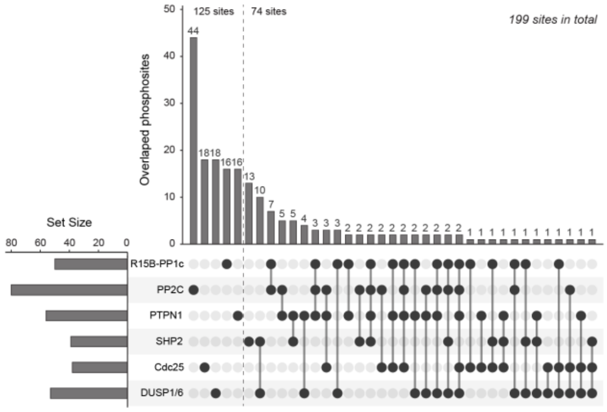

Figure S4. Upset plot showing the number of overlapped phosphosites that are up-regulated by treatment of each inhibitor.

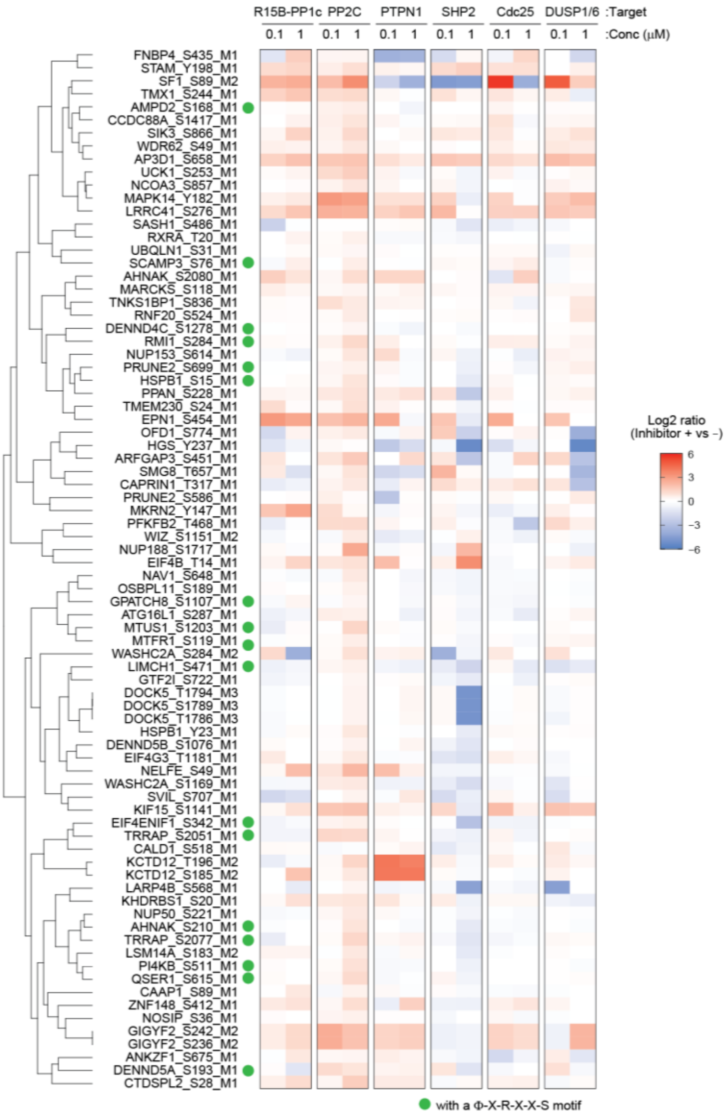

Figure S5. Heatmap showing the log2-transformed phosphosite abundance changes induced by inhibitor treatment.

Uncropped images of western blotting data

Figure S1

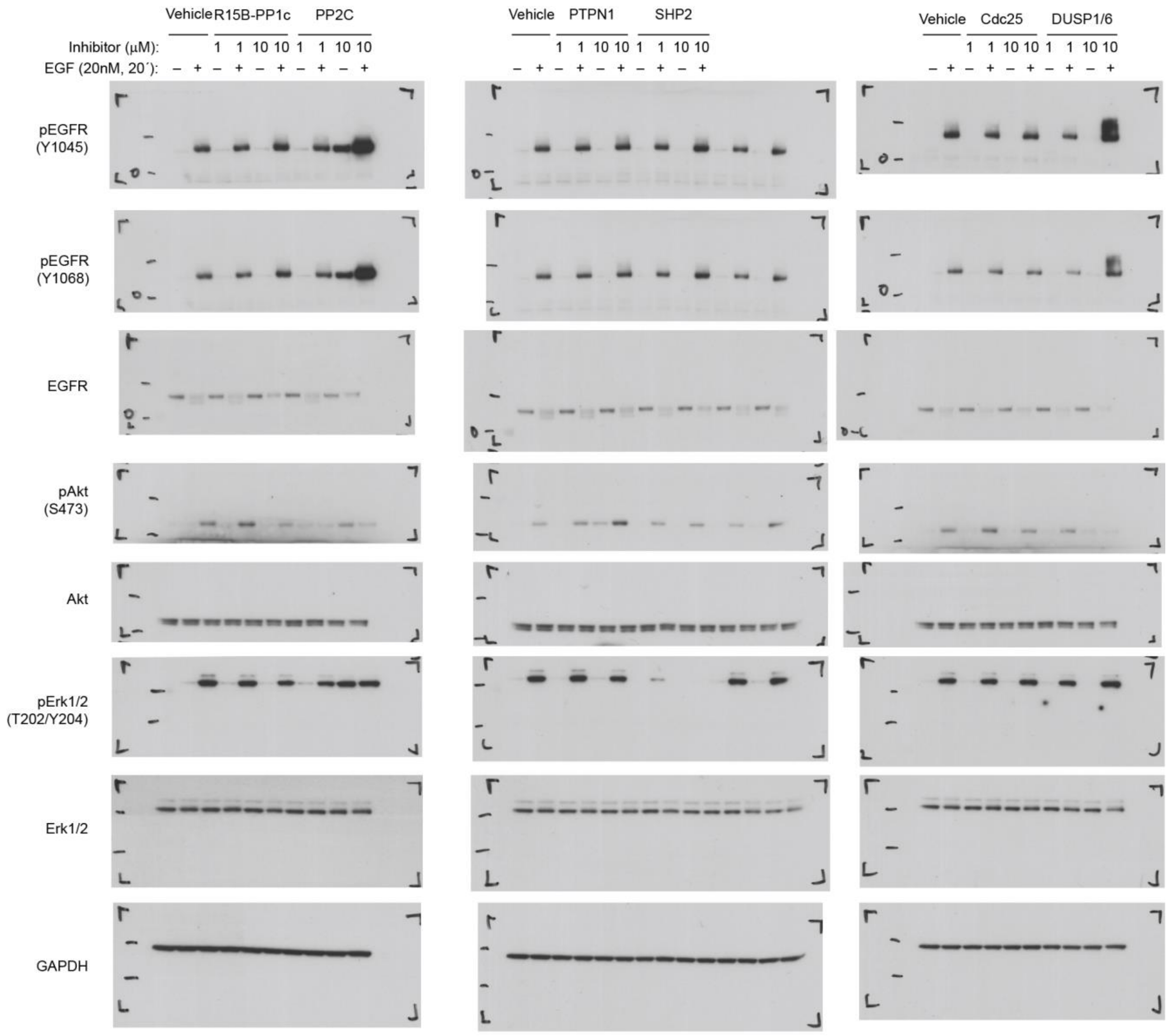

Figure 3G

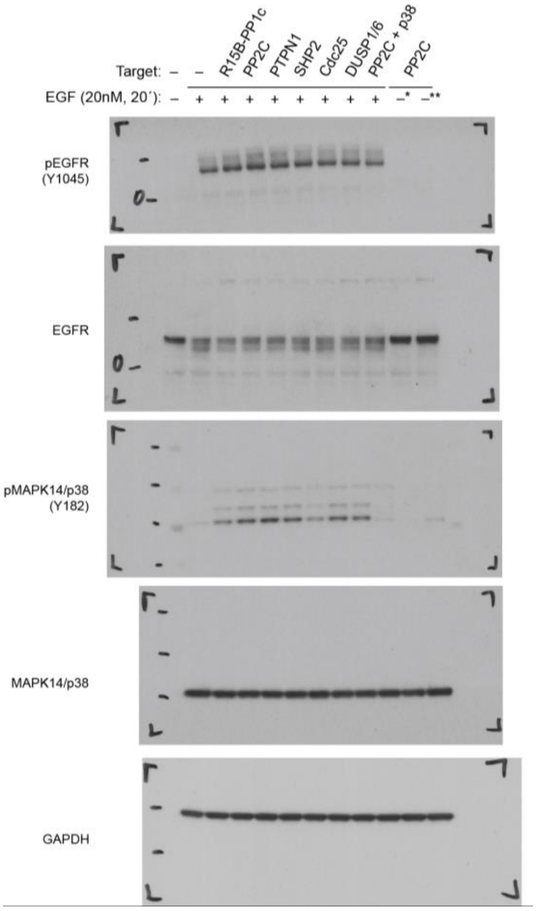

Target: – – R15B-PP1c PP2C PTPN1 SHP2 Cdc25 DUSP1/6 PP2C + p38 PP2C
EGF (20nM, 20´): – + + + + + + + + –* –**
pEGFR (Y1045)
EGFR
pMAPK14/p38 (Y182)
MAPK14/p38
GAPDH
